# Supplementary material for: Analysis and Reliability of Anthropometric Measurements during Pregnancy: A Prospective Cohort Study in 208 Pregnant Women
Source: J Clin Med. 2021 Aug 31;10(17):3933. doi: 10.3390/jcm10173933 (PMC8432171; doi:10.3390/jcm10173933)
Supplement: Supplementary file 1 [file jcm-10-03933-s001.zip › jcm-1329540-supplementary.pdf]

Supplementary material

Tabla S1: Gestational weight gain by trimester and by newborn sex

	Sexo	N	Media	Desviación típ.	p-valor
**Peso Ganado 1T - 2T**	Mujer	99	1,96	2,49	0,588
	Varón	82	2,15	1,93	
**Peso Ganado 1T - 3T**	Mujer	99	7,11	3,90	,215
	Varón	82	7,75	2,87	
